# Effectiveness of Working Memory Training among Subjects Currently on Sick Leave Due to Complex Symptoms

**DOI:** 10.3389/fpsyg.2016.02003

**Published:** 2017-01-06

**Authors:** Julie K. Aasvik, Astrid Woodhouse, Tore C. Stiles, Henrik B. Jacobsen, Tormod Landmark, Mari Glette, Petter C. Borchgrevink, Nils I. Landrø

**Affiliations:** ^1^Department of Circulation and Medical Imaging, Faculty of Medicine, Norwegian University of Science and TechnologyTrondheim, Norway; ^2^Hysnes Rehabilitation Center, St. Olav's University HospitalTrondheim, Norway; ^3^National Competence Centre for Complex Disorders, St. Olav's University HospitalTrondheim, Norway; ^4^Department of Public Health and General Practice, Norwegian University of Science of TechnologyTrondheim, Norway; ^5^Department of Psychology, Norwegian University of Science and TechnologyTrondheim, Norway; ^6^Clinical Neuroscience Group, Department of Psychology, University of OsloOslo, Norway

**Keywords:** adaptive working memory training, spatial working memory, inhibitory control, self-perceived memory functioning, complex symptoms

## Abstract

**Introduction:** The current study examined if adaptive working memory training (Cogmed QM) has the potential to improve inhibitory control, working memory capacity, and perceptions of memory functioning in a group of patients currently on sick leave due to symptoms of pain, insomnia, fatigue, depression and anxiety. Participants who were referred to a vocational rehabilitation center volunteered to take part in the study.

**Methods:** Participants were randomly assigned to either a training condition (*N* = 25) or a control condition (*N* = 29). Participants in the training condition received working memory training in addition to the clinical intervention offered as part of the rehabilitation program, while participants in the control condition received treatment as usual i.e., the rehabilitation program only. Inhibitory control was measured by The Stop Signal Task, working memory was assessed by the Spatial Working Memory Test, while perceptions of memory functioning were assessed by The Everyday Memory Questionnaire-Revised.

**Results:** Participants in the training group showed a significant improvement on the post-tests of inhibitory control when compared with the comparison group (*p* = 0.025). The groups did not differ on the post-tests of working memory. Both groups reported less memory problems at post-testing, but there was no sizeable difference between the two groups.

**Conclusions:** Results indicate that working memory training does not improve general working memory capacity *per se*. Nor does it seem to give any added effects in terms of targeting and improving self-perceived memory functioning. Results do, however, provide evidence to suggest that inhibitory control is accessible and susceptible to modification by adaptive working memory training.

## Introduction

Recent research shows that patients on sick leave report substantial problems with memory and attention (Aasvik et al., 2015). Such self-reported cognitive impairments are typically presented in relation with difficulties in social settings and everyday activities (Stenfors et al., 2013) and have been associated with work-related stress and reduced coping abilities (Broadbent et al., 1982; Folkmann et al., 1986). Sick leave is associated with a heterogeneous series of stress-related symptoms such as pain, fatigue, insomnia, depression and anxiety (Henderson et al., 2005). Patients with the aforementioned symptoms frequently report problems that go beyond the primary characteristics, such as reduced mental alertness, increased distraction, and problems with forgetfulness (Derousné et al., 1999; Mowla et al., 2008; Kronholm et al., 2009; Tesio et al., 2015). A wealth of studies have also found evidence of cognitive dysfunctions, the most consistent findings being related to impairments in working memory (WM) and executive functioning (Christopher and MacDonald, 2005; Derakshan et al., 2009; Constant et al., 2011; Fortier-Brochu et al., 2012; Landrø et al., 2013). Despite the impact and frequency of cognitive deficiencies, clinicians have primarily focused on treating the overarching symptoms i.e., the insomnia, the pain, the fatigue and so forth, with less focus given to treating the cognitive impairments.

WM has been implicated in a wide variety of complex cognitive and behavioral processes including selective attention, memory and learning, emotional regulation, and pain perception (Ochsner and Gross, 2005; Oosterman et al., 2010). Even very small differences in capacity may have huge consequences in various domains including academic achievement, occupational functioning and emotional health (Alloway and Alloway, 2010; Joormann and D'Avanzato, 2010; Oosterman et al., 2010; Alloway et al., 2013). Hence, examining if focused WM training improves cognitive function and perceptions of memory functioning is highly relevant in the rehabilitation of cognitive deficits.

WM is considered a mental workspace, and may be defined as the ability to actively hold and manipulate information in mind, while simultaneously filtering out or inhibiting distractions from entering the active state (Kane and Engle, 2000). Inhibitory control refers to the ability to resist or filter out distraction, and cancel irrelevant or no longer relevant responses (Miyake et al., 2000). According to the aforementioned definition of WM, inhibitory control is essential in guarding information processing in WM, protecting processing from irrelevant or distractive stimuli. Apart from the role inhibitory control plays in WM, it is also recognized as an underlying mechanism involved in almost all complex cognitive and behavioral processes.

When it comes to WM training, the main question is if effects of WM training extend beyond the specific tasks presented within the training program. Transfer of training effects is a debated topic, and although there are some promising results (Klingberg et al., 2002, 2005; Westerberg et al., 2007; Jaeggi et al., 2008; Brehmer et al., 2012; Salminen et al., 2012; Anguera et al., 2013; Au et al., 2015; Waris et al., 2015), some also report negative findings (Owen et al., 2010; Redick et al., 2012; Zinke et al., 2012). For reviews see (Klingberg, 2010; Shipstead et al., 2012; Melby-Lervåg and Hulme, 2013). The inconsistent results highlight the need to identify factors that may moderate transfer, and the nature of the underlying mechanisms through which transfer may occur. Because inhibitory control makes such an intrinsic and integral part of working memory, and because it also has the potential to act on a broader landscape of cognitive abilities, it may be defined as one of the unique mechanisms that promote transfer from WM training. In addition, an important but often somewhat neglected aspect of WM training is whether such training has the ability to improve subjective perceptions of cognitive functioning. Such self-perceptions may be just as relevant when considering the practical value of this type of intervention. Following this, an essential question is if WM training is better or more efficient in targeting this aspect than other cognitive treatment programs.

The current study aimed to examine if adaptive WM training improves objective cognitive functioning by strengthening performance on a test of spatial working memory and a test of inhibitory control. We also sought to examine if adaptive WM training improves subjective cognitive functioning by improving perceptions of everyday memory functioning. Our first hypothesis was that participants receiving adaptive WM training would perform better on post-tests of spatial working memory than the comparison group. Our second hypothesis was that participants who received adaptive WM training would demonstrate better response inhibition compared to the comparison group as demonstrated by significantly lower Stop Signal Reaction Time (SSRT). Our third hypothesis was that participants receiving adaptive WM training would report substantially less subjective memory complaints at post-testing than the comparison group.

## Materials and methods

The study was approved by the Regional Committee for Medical and Health Research Ethics (2013/634 REK-midt) and adhered to the Helsinki Declaration. All participants were given a complete description of the study and gave written informed consent before inclusion.

### Study design and setting

This was a randomized controlled experimental training study, with pre/post-test design. Participants were recruited from an inpatient vocational rehabilitation center in Norway. General practitioners referred patients who were on sick leave to a 3.5–week inpatient intervention at a vocational rehabilitation center. All participants answered a web-based survey before they met with a multidisciplinary team (physician, psychologist, and physiotherapist) at an outpatient clinic for further assessment according to inclusion criteria. The survey included measures of socio-demographics, pain, insomnia, fatigue, depression and anxiety. The collected data were used as source material by the outpatient/inpatient clinics and also included in a research database.

Participants were randomly assigned to either the training condition or the control condition. Groups of maximum 16 subjects were admitted at each time point; to avoid cross-contamination every other group was offered adaptive WM training. This design was specifically chosen in order to encourage continued effort and motivation among the participants in the training condition. Participants assigned to the experimental condition received adaptive WM training (Cogmed) for 5 weeks in addition to the clinical intervention offered by the vocational rehabilitation center. Participants assigned to the control condition received only treatment as usual (i.e., the vocational rehabilitation program). Participants were assessed individually at the University Hospital at two different time points: Prior to training/stay at the vocational rehabilitation center (T1) and within 3 weeks of completing their training/their stay at the vocational rehabilitation center (T2).

The participants in the comparison group were offered WM training after study completion; this was done to reduce any disappointment and/or lack of motivation due to being randomized to the control condition.

As part of the pre-and post-test design all participants were given the Stop Signal Task (SST), and the Spatial Working Memory test (SWM) from the Cambridge Neuropsychological Test Automated Battery (CANTABeclipse, 2012). They also answered a self-report questionnaire assessing everyday memory failures, The Everyday Memory Questionnaire-Revised (Royle and Lincoln, 2008).

Subjects were excluded only if they were diagnosed with severe mental disorders (acute psychosis, ongoing manic episode, or suicidal ideation) ongoing substance abuse or if they had any neurological traumas or illnesses.

### Participants

**Figure 1:**
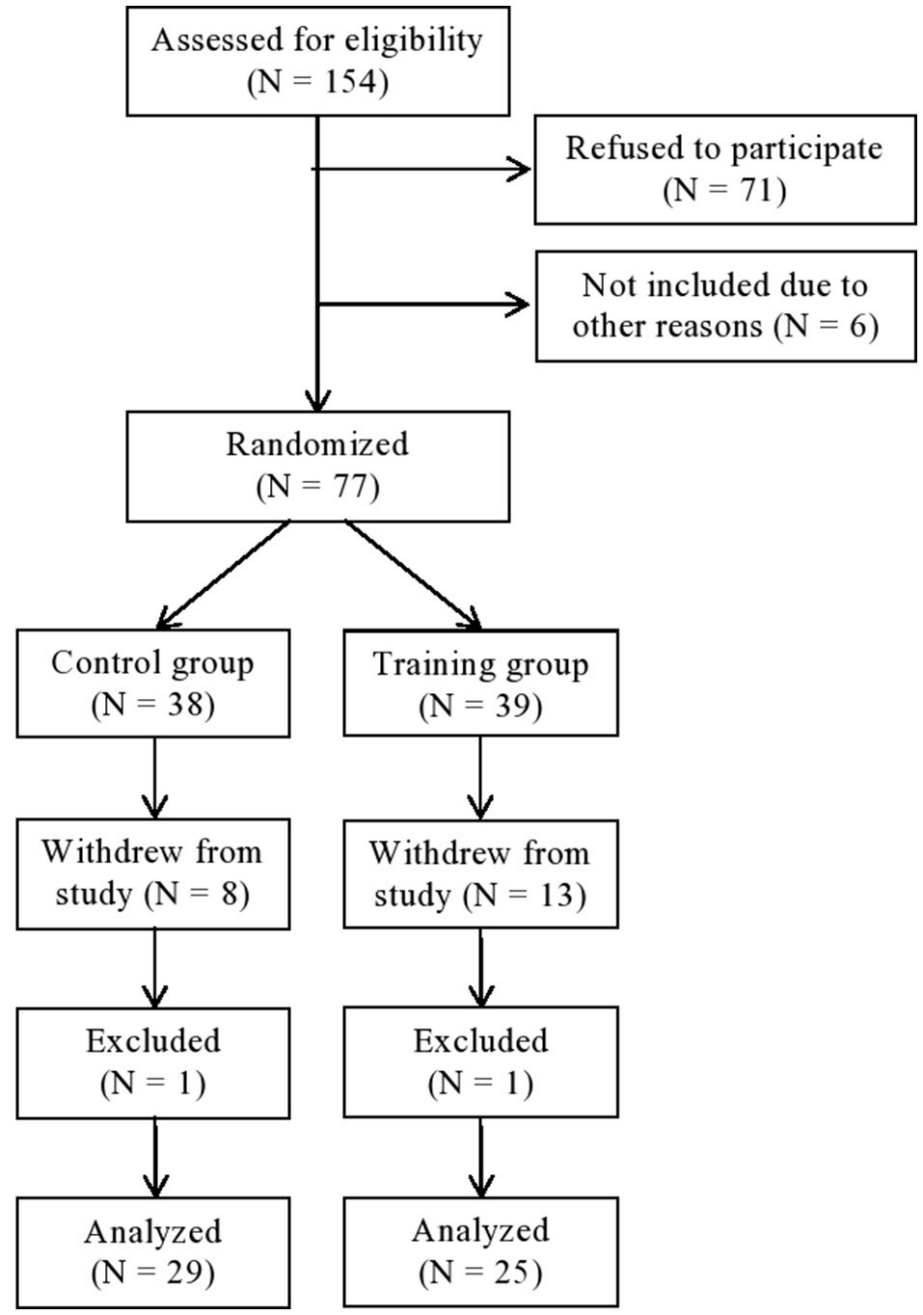
**Consort flow chart**.

Between October 2013 and April 2015, 148 patients were asked to participate in the study (see Figure 1). A total of 77 participants initially volunteered to take part in the study. Those who declined did so because they worried that the WM training would be too demanding and thus chose to focus on the vocational rehabilitation program. A total of 21 participants (27.3 %) withdrew from the study, 13 subjects from the training condition and 8 subjects from the control condition. In addition, due to malfunction in the CANTAB software, four participants did not complete their testing on the SWM and/or the SST test. When extracting those who withdrew from the study, we were left with a total of 54 subjects (44 females and 10 males). Mean age of the cohort was 43.3 years (*SD* = 10.6). All participants were on sick leave (> 8 weeks) due to symptoms of pain, fatigue, insomnia, depression and anxiety. All of the participants experienced a combination of two or more of the listed symptoms.

### The occupational rehabilitation program

All subjects participated in a standardized occupational rehabilitation program, based on acceptance and commitment therapy (ACT) (Hayes et al., 2003). Each participant was assigned to a return to work coordinator that followed them during their stay. The program included a combination of group treatment (8 participants per group, 8 sessions, total of 16 h) and individual approaches (5 sessions, total of 5 h). The program offered sessions of mindfulness (7 sessions, for a total of 3.5 h) and educational lectures on specific topics such as stress and stress management, nutritional diet, pain, and physical activity (4 lectures, total of 6.5 h). The program included physical training (10 sessions, total of 12 h), focusing on endurance, strength, and mobility. Individual exercises were adjusted according to the need of each participant, led by physical therapists. All subjects adhered to the rehabilitation program. All coordinators were trained and supervised in ACT, recordings was included to assure treatment fidelity. The program was scheduled for 7 h each weekday for a total 17 days not counting weekends. Details on the rehabilitation program can be found elsewhere (Fimland et al., 2014).

### The adaptive WM training intervention

The adaptive WM training was implemented by the use of a commercial software product (Cogmed QM). We chose this program because it is a well-studied WM training program, it offers adaptive WM training which is essential when examining if such training has the potential to improve WM capacity and inhibitory control. Upon receiving a username and a password, the participants may perform the training at any chosen computer, lap-top or iPad as long as it has internet access. The program consists of 12 different WM training exercises designed to train spatial and verbal-numeric WM, and inhibitory control (for a detailed description of the different exercises see Cogmed QM, www.cogmed.com). Participants have to complete one training session every weekday for 5 weeks. Each session consists of eight different tasks each designed to train different aspects of WM. It takes ~30–45 min to complete one session. In the first training session all participants start at the same level, the task being to remember two items. Task difficulty is continuously adjusted according to each individual's performance to ensure that participants perform at the limit of their ability, in order to progressively increase WM capacity. Difficulty is adjusted by either increasing or decreasing the number of items that need to be processed in WM, such that each individual obtains a 60% accuracy on each task in every session. In the following training sessions participants start at the level they reached in the previous session. Participants started their training at home, one and a half week prior to their stay at the rehabilitation center. This was done to allow participants to simultaneously complete their Cogmed training and their stay at the rehabilitation center.

Participants were given feedback about their training by telephone, or by meetings. The feedback was related to motivational aspects and also to their performance in terms of progression as assessed by the indexes offered by the Cogmed program: The Start Index, the Max Index and the Index Improvement.

The Cogmed program provides compliance measures describing the number of training sessions and a quantified measure of compliance with the training program referred to as Cogmed Improvement Index score. The Cogmed Improvement Index score is computed automatically based on two training indices: The Start Index and the Max Index. The Start Index is based on results from day 2 to 3, and the Max Index is based on the best results obtained during the training period. The Improvement Index is calculated by subtracting the Start Index from the Max Index. The mean improvement index for individuals aged 18–65 years is *M* = 29 (normal range 15–41), higher Index scores indicate good compliance and effort with the training. For further details about the training intervention and the training algorithm see Cogmed QM; www.cogmed.com or Klingberg et al. (2002). Table 1 present's data describing the number of completed training sessions, time spent on training and the different index scores. The mean training time for each session were *M* = 33.3 min. Subjects completed on average 23 training sessions and the mean Improvement Index of *M* = 24 indicated good compliance and effort.

**Table 1:** **Cogmed indexes given in means and standard deviations**.

**Cogmed indexes**	***N***	**Mean**	***SD***
Number of training sessions	25	23	2.5
Time spent on each session	25	33.3	4.5
Start Index	25	82.8	8.7
Max Index	25	106.5	12.2
Index Improvement	25	24.0	7.5

### Assessments

#### Memory complaints

Subjective cognitive complaints were based on a self-report questionnaire, The Everyday Memory Questionnaire-Revised (EMQ-R) (Royle and Lincoln, 2008). The EMQ was originally developed by Sunderland et al. (1983) and later revised (shortened) by Royle and Lincoln. The EMQ-R consists of 13 items, each item is rated on a 5-point scale ranging from A, scored as zero “Once or less in the last month,” to E, scored as four “Once or more in a day.” The items were summed, giving a scale from 0 to 52. Reliability tests on the EMQ-R have shown a strong internal reliability, with a Cronbach's alpha score of 0.89 (Royle and Lincoln, 2008).

#### Pain

We used three items from The Brief Pain Inventory (BPI) for measuring pain intensity (Cleeland and Ryan, 1994). The items ask responders to rate their worst, least and average levels of pain. Each item is rated on a 10-point scale ranging from 0–“no pain” to 10–“pain as bad as you can imagine.” In validation studies the BPI has shown good psychometric properties (Klepstad et al., 2002).

#### Fatigue

Fatigue was measured with The Chalder Fatigue Questionnaire (Chalder et al., 1993), which consists of eleven questions, reflecting physical and mental fatigue. Each item has four response categories that are scored bimodally 0-0-1-1. Responses were summed in a scale from 0 to 11. The scale has been validated for a Norwegian population (Loge et al., 1998).

#### Depression and anxiety

The Hospital Anxiety and Depression Scale (HADS) was used to assess symptoms of anxiety and depression (Zigmond and Snaith, 1983). The fourteen item scale is divided into two sub-scales; one sub-scale scores depression and the other scores anxiety. Each item ranges from 0 to 3, and the items are summed, giving a scale ranging from 0 to 21 in each sub-scale. The psychometric properties of the scale have been validated in various populations, as well in the Norwegian general population (Bjelland et al., 2001; Olssøn et al., 2005).

#### Insomnia

The Insomnia Severity Index (ISI) (Bastien et al., 2001) was used to measure insomnia. This is a self-report questionnaire consisting of seven items designed to assess insomnia severity by the following: Difficulty falling asleep, night time awakenings, early morning awakenings, impairment of daytime functioning due to sleep disturbances, noticeability of problems, distress or worry caused by sleep disturbances, and dissatisfaction with sleep. Each item is rated according to a 5-point scale, ranging from 0 (not at all) to 4 (very much), giving a scale of 0–28. The psychometric properties of the instrument in terms of detecting insomnia cases have been determined (Morin et al., 2011).

#### General cognitive functioning

General cognitive functioning was estimated from scores on a subtest from the WAIS-III: Picture completion (PC) (Wechsler, 1997). The test consists of 25 cards and each card displays a picture where a part is missing. The subject is told to identify the missing part, within a time limit of 20 s. It has been found to give a reliable and valid measure of cognitive functioning.

#### Inhibitory control

The Stop Signal Task (Logan and Cowan, 1984; Logan et al., 1984) (SST) provides a measure of response inhibition, the Stop Signal Reaction Time. A white ring containing a left or right pointing arrow is shown on the screen. First, the subjects are instructed to press on the corresponding (left/right) button on a press pad as soon as they see the direction of the arrow. They are given a trial to practice the instruction. In the second condition of the task, the subjects are told to press on the corresponding button like before, but should avoid pressing the button whenever hearing a beep (the Stop Signal). By varying the timing of the Stop Signal Delay (SSD) throughout the test, the program regulates the probability of stopping such that stopping occurs approximately 50% of the time for each subject. The Stop Signal Reaction Time is calculated by subtracting the SSD from the median GO Reaction Time (the reaction time on trials with no Stop Signal). The Stop Signal Reaction Time (SSRT) (last half) was chosen as the main outcome measure. The chosen outcome reflects the ability to inhibit a dominant response.

#### Working memory

Spatial Working Memory (SWM) measures the subject's ability to retain, manipulate, and update spatial information in working memory (Owen et al., 1990; Sahakian and Owen, 1992). The task also provides an assessment of the heuristic strategy. The screen displays a number of colored boxes. The subjects are instructed to find a hidden token in each of the boxes, and use them to fill up an empty column on the side of the screen. Returning to a box where a token has already been found represents an error. The test starts with three boxes, then four, six, and finally eight boxes. The pattern and color of the boxes are changed in each trial. The outcome of interest was the measure of strategy. The outcome reflects the ability to adopt an efficient strategy to complete the task.

### Statistical analyses

Data was analyzed using the Statistical Package for the Social Sciences (SPSS version 20.0; 185 IBM Corporation, Armonk, NY, USA). Descriptive statistics are given as means and standard deviations for continuous variables and as numbers and percentages for categorical variables. Within group comparisons of performance on the tests of spatial working memory, inhibitory control and self-perceived memory functioning from pre-to post-testing were obtained using paired samples *t*-test. To test our hypotheses, we used fixed effect analyses of covariance (ANCOVA) with post-test (SSRT-last half, SWM-strategy and EMQ-R) performance as the dependent variable, with group a fixed factor, and pre-test performance as a covariate. Because age and gender might be confounders, we also adjusted for these variables in our analyses. On the SST test, we excluded three scores due to extreme values (outliers). Values above 300 ms and below 80 ms were determined as extreme values based on prior knowledge and experience with this task paradigm. The significance level was set at *p* < 0.05. We performed a one-tailed Spearman correlation analysis to examine if the training induced improvement in WM capacity (reflected by the Cogmed index improvement) was associated with the improvement in inhibitory control (SSRT-last half).

## Results

Descriptive data including self-reported levels of pain, insomnia, fatigue, depression, anxiety and everyday memory failures are presented in Table 2. The experimental group consisted of 25 subjects (21 females and 4 males) and the control group consisted of 29 subjects (23 females and 6 males). There was some difference in age between the two groups. Analyses confirmed that the difference in age was not statistically significant (*p*-value = 0.065). The comparison group had been on sick leave for an average of 15 months (*M* = 15 months), while the training group had been of on sick leave for an average of 14 months (*M* = 14 months). The groups were similar in general cognitive ability; they reported similar levels of pain, insomnia, fatigue, depression, and anxiety, all within what is considered subclinical range. Both groups reported considerable memory problems. Their scores were higher than what has been found in MS and stroke patients (Royle and Lincoln, 2008).

**Table 2:** **Descriptive data**.

**Descriptive data**	**Training group**			**Control group**		
	***N***	***M***	***SD***	***N***	***M***	***SD***
Gender: Females (%)	21 (84)			23 (79)		
Age	25	46.2	10.1	29	40.8	10.5
Duration of sick leave (months)	25	13.8	11.2	29	15.4	15.6
WAIS-picture completion (max score 25)	25	23.0	1.4	29	22.8	1.8
BPI-maximum pain (scale 0–10)	24	5.0	2.5	25	5.8	2.8
BPI-least pain (scale 0–10)	23	2.3	1.9	25	2.6	1.7
BPI-average pain (scale 0–10)	24	4.0	2.5	25	4.0	2.0
ISI-Insomnia (scale 0–28)	24	12.7	5.7	26	12.6	5.5
CFS-Fatigue (scale 0–11)	24	2.2	2.4	26	2.2	2.4
HADS-Depression (scale 0–21)	24	7.3	2.9	26	7.5	3.5
HADS-Anxiety (scale 0–21)	24	8.1	3.1	26	8.9	3.1

*WAIS, Wechsler Adult Intelligent Scale used as a measure of general cognitive ability; BPI, Brief Pain Inventory; ISI, Insomnia Severity Index; CFS, Chalder Fatigue Scale; HADS, Hospital Anxiety and Depression Scale*.

Pre/post means, standard deviations and paired samples *t*-tests of main outcome variables are presented in Table 3. The results indicated a substantial time reduction in SSRT-last half in the experimental condition (*p*-value = 0.033) but not in the control condition (*p*-value = 0.973). The correlated *t*-tests indicated no statistically significant changes in SWM strategy from pre to post-tests in either of the groups. Both groups demonstrated significantly less memory problems at post-testing.

**Table 3:** **Descriptive data and *t*-tests of outcome variables**.

**Group**	**Tests**	**Pre-test**			**Post-test**			***t***	**Sign (*p*-value)**
		***N***	**Mean**	***SD***	***N***	**Mean**	***SD***		
Control group	SWM Strategy	26	30.1	6.5	25	30.6	7.2	0.3	0.768
	SSRT (last half)	28	171.8	32.6	26	171.1	37.1	−0.03	0.973
	EMQ-R	29	21.8	12.7	28	16.6	9.6	3.1	0.004
Training group	SWM Strategy	24	30.3	5.6	24	28.7	6.7	1.4	0.187
	SSRT (last half)	22	178.1	28.7	25	168.7	43.4	2.2	0.033
	EMQ-R	25	21.9	10.3	25	15.3	10.7	3.7	0.001

*SWM, Spatial Working Memory; SSRT, Stop Signal Reaction Time; EMQ-R, Everyday Memory Questionnaire–Revised*.

As presented in Table 4A, the ANCOVA showed no statistically significant difference between the two groups on the post-test measure of SWM Strategy. Hence, the Cogmed training did not translate into better performance on SWM post-test relative to the comparison group. Neither was there an effect of age, gender or group by gender. As presented in Table 4B, the ANCOVA showed a significant training effect on post SSRT-last half. The subjects in the training condition performed considerably better than the subjects in the control condition on post-tests of inhibitory control (*p*-value = 0.025). The effect measure shows an improvement among the subjects in the training condition relative to the subjects in the control condition. Pre-test scores on SSRT-last half and age co-varied with post-test SSRT scores. There was an effect of age, but there was no effect of gender, nor was there a group by gender effect. We did a Spearman Correlation analysis to examine if the improvement in WM after Cogmed training (index improvement, see Table 1) was associated with the change/improvement in the post-test of inhibitory control (SSRT), we found a significant correlation (0.386), *p*-value 0.035 between the two measures. As presented in Table 4C, the ANCOVA showed no significant group effects on post scores on the EMQR. In addition, there was no effect of age, gender, or group by gender.

**Table 4A:** **ANCOVA analysis showing group effect on measure of Spatial Working Memory Strategy**.

**Variable**	**β-estimate**	***95%*** **CI**	***F***	**Sign (*p*-value)**
Control vs. Training	0.968	−5.836–7.772	0.578	0.448
Pre-test SWM Strategy	0.825	0.584–1.066	47.6	0.000
Age	0.004	−0.139–0.146	0.003	0.959
Women vs. Men	0.766	−5.162–6.695	0.436	0.513
Group^*^Gender	0.888	−6.701–8.477	0.056	0.815

*Adjusted R squared = 0.540. SWM Strategy refers to Spatial Working Memory Strategy*.

**Table 4B:** **ANCOVA analysis, showing group effect on measure of inhibitory control (SSRT-last half)**.

**Variables**	**β-estimate**	***95% CI***	***F***	**Sign (*p*-value)**
Control vs. Training	40.620	0.644–80.595	5.375	0.025
Pre-test SSRT (last half)	0.570	0.274–0.866	15.144	0.000
Age	1.046	0.145–1.946	5.499	0.024
Women vs. Men	20.699	−13.465–54.863	0.301	0.586
Gender^*^Group	−29.166	−73.946–15.614	1.730	0.196

*Adjusted R squared = 0.323. SSRT (last half): Stop Signal Reaction Time used as a measure of inhibitory control*.

**Table 4C:** **ANCOVA analysis showing group effect on measure of self-perceived memory functioning (EMQ-R)**.

**Variables**	**β-estimate**	***95% CI***	***F***	**Sign (*p*-value)**
Control vs. Training	0.554	−9.499–10.607	0.164	0.687
Pre-test EMQ-R	0.597	0.404–0.790	38.583	0.000
Age	0.041	−0.180–0.262	0.139	0.711
Women vs. Men	−0.747	−9.308–7.813	0.004	0.952
Group^*^Gender	2.595	−9.976–12.281	0.043	0.836

*Adjusted R squared = 0.414. EMQ-R Everyday Memory Questionnaire–Revised used as a measure of self-perceived memory functioning*.

## Discussion

The present study examined the effects of a 5 week adaptive WM training program (Cogmed) in a group of patients currently on sick leave due to symptoms of pain, insomnia, fatigue depression and anxiety. Training effects were assessed by comparing performance on three tasks assessing spatial working memory, inhibitory control (Stop Signal Reaction Time), and perceptions of everyday memory functioning. We found no evidence in support of our first hypothesis, stating that participants in the training condition would perform better than the comparison group on the post-test of spatial working memory. We did, however, find support for our second hypothesis, stating that participants who received WM training would perform better than the comparison group on post-tests of inhibitory control. Moreover, we found no support for our third hypothesis stating that participants who received WM training would report substantially less memory problems compared to participants in the comparison group.

While both the Cogmed program and SWM test target spatial working memory, they do it in slightly different ways. Although somewhat speculative at this stage, it may be that the somewhat different test designs may help explain the discrepancy in results with regard to the test of SWM and the test of inhibitory control. The Cogmed program consists of several spatial working memory tasks with different designs. One of the tasks, named “chaos” involves keeping track of an increasing number of specific objects presented within a larger matrix of objects. All of the objects move and continuously change positions. The program signals the objects that have to be remembered by briefly changing their color. Because only a few of the objects have to be remembered, the remaining objects act like distractors which must be blocked or inhibited to keep track of the selected objects. The “chaos” task therefore targets both SWM and the ability to block or inhibit distractors. As the number of items that has to be remembered increases, the task requires more effortful control of attention thereby putting greater demands on inhibitory control. The SWM test involves keeping track of a single object, which shifts position among an increasing number of stationary objects. Because the objects do not move, they might not be as intrusive or potent distractors as if they had moved. Thus, while the SWM test measures the ability to maintain spatial information in WM, it does not make the same requirements for inhibitory control and therefore to a lesser degree, measures the ability to inhibit distractive stimuli. Hence, while the chaos task may have trained inhibitory control, the improved ability to inhibit distractors is not as targeted within the SWM test paradigm, and this may explain why the improved performance in inhibitory control did not translate into better performance on the SWM task. Our results may suggest that inhibitory control is more accessible and susceptible to transfer of training effects than SWM *per se*, future research should examine if this is indeed the case.

Because inhibition in and by itself has broad implications across various cognitive and behavioral processes including perceptual and emotional regulation, this result warrants attention (Ochsner and Gross, 2005; Joormann, 2010; Oosterman et al., 2010; Miyake and Friedman, 2012). There was a difference between the groups in regard to the amount of treatment that they received. While the training group received WM training in addition to the rehabilitation program, the control group only received the rehabilitation intervention. As a result we cannot exclude the possibility that the difference in treatment intensity might have had some effect in terms of the improved inhibitory control. However, because we found a significant correlation (*p*-value = 0.035) between the Cogmed Index Improvement and the improvement in inhibitory control (SSRTs) it indicates that the WM training was indeed related to the improvement in inhibitory control. Still, the present finding should be considered as a preliminary result and must be replicated by other studies. When it comes to the improvement in inhibitory control (SSRT), it might seem modest. however, it might be relevant in situations that require a high level of attentional focus and rapid decision-making in which the ability to quickly inhibit or filter out distracting information is crucial such as in a complex work environment, or in terms of strengthening the ability to regulate and control impulsive and/or compulsive behaviors (Dalley et al., 2011).

The negative finding with regard to perceptions of everyday memory functioning might reflect that the clinical intervention offered to both groups had a strong effect on this specific measure. Alternatively, it might reflect a “feel good” or an expectancy effect related to the interventions. Some studies have reported that subjective memory problems are associated with objective cognitive impairments (Grace et al., 1999; Landrø et al., 2013; Tesio et al., 2015). It seems reasonable to assume that the improvements in inhibitory control following training (such as in our study) would also generate a greater reduction in self-reported memory problems, but this was not the case. A number of studies have failed to find any association between self-reported and objective measures of memory impairments (Suhr, 2003; Glass et al., 2005). The lack of association may reflect that self-report questionnaires and neuropsychological tests relate to different concepts, tapping into different sources and facets of information (Williams et al., 2011). While self-report questionnaires may reflect metacognitive beliefs and metacognitive monitoring (Jacobsen et al., 2016), such subjective information typically lies beyond the scope of neuropsychological tests, such as the SST test.

Several studies have examined effects of WM training on measures of inhibition (Klingberg et al., 2002, 2005; Olesen et al., 2004; Westerberg et al., 2007; Thorell et al., 2009; Chein and Morrison, 2010; Van der Molen et al., 2010; Dahlin, 2011; Anguera et al., 2013; Berkman et al., 2014). This specific field is characterized by extensive cross-study variability; focusing on different subgroups (i.e., children and young adults with and without ADHD, students, elderly, healthy, stroke patients, etc.), using different tests to measure inhibition (Stroop, go/no go tests etc), and finally using different training protocols (Cogmed, OddYellow, Neuro Racer etc.). All of the aforementioned factors complicate and confuse attempts to generalize results. The mixed findings underscore the fact that training effects may vary due to specific characteristics such as age, neurological, psychological and cognitive factors. As mentioned above, studies differ in regards to which tests they have used to tap inhibition. The Stroop task has been used in several studies (Klingberg et al., 2002, 2005; Olesen et al., 2004; Westerberg et al., 2007; Thorell et al., 2009), but due to flaws in the administration of the test (not including congruent trials) some of those studies (Klingberg et al., 2002, 2005; Olesen et al., 2004) have been criticized because performance cannot be interpreted as related to inhibitory control (Shipstead et al., 2012). Still, some studies have found promising results in terms of improved inhibitory control (Chein and Morrison, 2010; Anguera et al., 2013; Berkman et al., 2014). Because training protocols vary in regards to task design, the number of tasks, and the intensity and length of training, results stemming from one training program may not be readily replicated by studies using different training protocols (Morrison and Chein, 2011). The mixed results may reflect that different WM training programs (more or less effectively) target different mechanisms in different subgroups depending on the neuropsychological profile of each group.

Recent theories and research suggest that deficiencies in inhibition may contribute to the development and maintenance of symptoms such as pain, insomnia, fatigue, depression and anxiety (Ochsner and Gross, 2005; Derakshan et al., 2009; Joormann, 2010; Joormann and D'Avanzato, 2010; Miyake and Friedman, 2012). In a fairly recent study researchers were able to demonstrate a direct link between pain sensation and cognitive inhibition; better inhibitory control was associated with lower pain sensitivity (Oosterman et al., 2010). Our results indicate that inhibition may be modulated and improved by adaptive WM training. Targeting inhibitory control may thus offer considerable promise in terms of improving the effectiveness of traditional therapeutic interventions (Siegle et al., 2007).

## Limitations

Our sample included a series of stress related and comorbid symptoms, all of which have been associated with impairments in WM. Although the WM profile might be similar across symptoms, there might be differences in terms of how the different symptoms affect the ability to profit from the WM training. The effect of these symptoms with regard to transfer effects could not be examined due to lack of statistical power. Future studies should address this aspect as it might be an important factor that moderates the potential to benefit from such training. Moreover, the duration and sensitization of different pain and fatigue states may not be linked to the duration of sick leave, making it difficult to sample a homogenous group of participants. We did not include tests to assess verbal-numeric WM although this was targeted by the training program. This study is limited by the fact that we did not include additional transfer measures for each of the cognitive domains that we examined. Furthermore, because the post-tests were conducted within 3 weeks of completing the training program the current results therefore reflect short-term effects. While this study shows an improvement in inhibitory control following training, this is only at test level and it may not transfer to real life.

## Conclusions

WM training was associated with improved inhibitory control as indicated by a statistically significant reduction in SSRTs among subjects in the training condition compared to the control condition. WM training did not improve performance on the SWM transfer measure. Self-perceived memory functioning did not differ between groups at post-testing. Improving inhibitory control may support working memory by strengthening the ability to quickly suppress or block distractive stimuli.

## Author contributions

All authors listed, have made substantial, direct and intellectual contribution to the work, and approved it for publication.

### Conflict of interest statement

The authors declare that the research was conducted in the absence of any commercial or financial relationships that could be construed as a potential conflict of interest.
